# *The Plant Cell* welcomes 2026 Assistant Features Editors

**DOI:** 10.1093/plcell/koag002

**Published:** 2026-01-08

**Authors:** Nancy A Eckardt, Mary Williams, Pablo A Manavella

**Affiliations:** American Society of Plant Biologists, USA; American Society of Plant Biologists, USA; Institute of Subtropical and Mediterranean Horticulture, University of Málaga, Spanish National Research Council (CSIC), 29010 Málaga, Spain

*The Plant Cell* is pleased to announce our Assistant Features Editors (AFEs) for 2026 (Fig. 1). *The Plant Cell* AFE program provides AFEs with experience in science writing for a broad audience, training in the peer-review process, and networking opportunities with our editorial board, authors, and other AFEs. The AFEs, in turn, deliver valuable service to the journal, our authors, and the scientific community by contributing “In Brief” article highlights and participating in editorial board activities. The AFEs gain experience and mentoring to improve their writing, receive editor and peer-review training, participate in editorial board meetings, and engage in other journal-related activities. In June of 2026, the AFEs will be supported to attend Plant Biology 2026 in Ottawa, Canada. Congratulations to our successful applicants! We are excited to work with this terrific group of early-career researchers in 2026 (Fig. 1).

**Figure 1.**
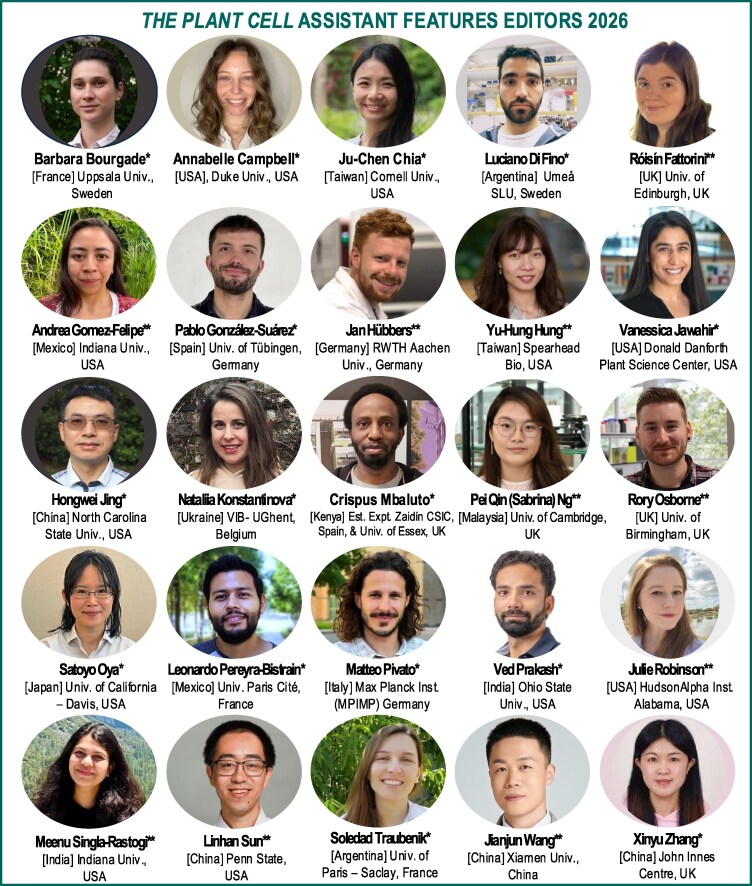
*The Plant Cell* Assistant Features Editors for 2026. [Country of Origin], Current affiliation. *Joining January 2026. **Continuing. Rory Osborne is continuing for a 3rd year as Senior AFE to assist with management of the AFE program.

*The Plant Cell* AFE program is highly competitive and a great addition to your C.V. We emphasize the writing samples and other application materials to provide evidence that an applicant shows a commitment to science communication, an aptitude for storytelling and writing in an engaging manner, and the ability to easily grasp the major findings of work published in *The Plant Cell*. We also seek to bring on a diverse group that collectively can cover the range of topics published in the journal. We accept applications for the AFE program every 2 years. The next application deadline for *The Plant Cell* AFE program will be in September 2027 for positions to start in 2028 and 2029. Our sister journal *Plant Physiology* will issue a call for applications for the *Plant Physiology* AFE program in 2026 for positions to start in 2027 and 2028. Watch for information on the journal websites and social media channels.

Our sincere thanks to the AFEs stepping down in 2026 for their years of service to the journal:

Laura Arribas-Hernández. IHSM La Mayora (CSIC-UMA), Málaga, Spain.

Leonard Blaschek. Umeå Plant Science Center, Department of Forest Genetics and Plant Physiology, Swedish University of Agricultural Sciences, Sweden.

Sonhita Chakraborty. Life Science Editors and artbysonhita, Toronto, Canada.

Vicky Howe. University of New South Wales, Australia.

Min-Yao Jhu. University of Cambridge, UK.

Nitin Uttam Kamble. IISER Thiruvananthapuram, India.

Gwendolyn Kirschner. James Hutton Institute, UK.

Renuka Kolli. University of Cambridge, UK

Guy Levin. Technion-Israel Institute of Technology, Israel

Christian Lorenzo. VIB-UGent, Belgium.

Regina Mencia. Instituto de Agrobiotecnología del Litoral-UNL-CONICET, Argentina

Margot Raffeiner. Ruhr University Bochum, Germany.

Shanice Webster. Duke University, USA.

Andrew Willoughby. Duke University, USA and Salk Institute for Biological Studies, USA

It has been a pleasure working with all of you and meeting in person those who could make the trip to the editorial board meeting and Plant Biology 25 in Milwaukee, WI, in July 2025. We wish you all the best in your future endeavors.

On a final note, Nancy (Nan) Eckardt will retire from her role as Director of *The Plant Cell* Features Editor program at the end of 2025. Nan has managed the AFE program since its inception in August 2017 and has greatly enjoyed working with the nearly 100 early-career researchers who served as AFEs in the last 8.5 years. Features Editor Mary Williams will take over management of *The Plant Cell* AFE program in 2026, with assistance from Senior AFE Rory Osborne.

## Data Availability

No new data are associated with this article.

